# In-ovo imaging using ostrich eggs: biodistribution of F-18-FDG in ostrich embryos

**DOI:** 10.3389/ebm.2025.10560

**Published:** 2025-06-19

**Authors:** Thomas Winkens, Pauline Schweitzer, Olga Perkas, Christian Kühnel, Ferdinand Ndum, Marta Pomraenke, Julia Greiser, Martin Freesmeyer

**Affiliations:** ^1^ Clinic of Nuclear Medicine, Jena University Hospital, Jena, Germany; ^2^ Working Group for Translational Nuclear Medicine and Radiopharmacy, Clinic of Nuclear Medicine, Jena University Hospital, Jena, Germany

**Keywords:** in-ovo imaging, ostrich eggs, animal model, glucose metabolism, preclinical imaging

## Abstract

In-ovo imaging using ostrich eggs has been described as an alternative to animal testing using rodents. This approach is not considered an animal experiment and it does not require small-animal imaging devices as ostrich eggs provide good image quality on regular CT, MRI or PET used in humans. The aims of this study were 1) to describe methods of radiopharmaceutical injection, 2) to explore normal biodistribution of F-18-FDG during a 60-min list-mode-PET/CT examination and 3) to compare biodistribution in-ovo to existing literature considering chicken and rodents. Vessel access was successful in 54/78 ostrich eggs. Highest FDG-uptake was observed in epiphyseal plates (0.36 ± 0.06 IA%/g; range 0.29–0.48 IA%/g) and brain (0.25 ± 0.05 IA%/g; range 0.21–0.36 IA%/g). *In-vivo* activity distribution on PET and *ex-vivo* activity distribution (well counter) showed comparable results (Spearman’s Rho range 0.795–0.882). No significant differences were observed regarding previous isoflurane exposure. Normal biodistribution of F-18-FDG in ostrich embryos using a standard PET/CT system for humans was mainly found as expected with highest uptake in epiphyseal plates and brain which is comparable to results on rodents and chicken embryos. Isoflurane anesthesia did not reveal significant differences regarding organ uptake. The results of this normal distribution study allow for interpretation of future disease models (inflammation, tumor) in ostrich embryos using F-18-FDG as radiopharmaceutical.

## Impact statement

This work shows that normal biodistribution of F-18-FDG in ostrich embryos is comparable to chicken embryos, rodents and humans. Thus, in-ovo-imaging using ostrich embryos represents a promising alternative to reduce animal research using rodents.

## Introduction

Recently, preclinical imaging using ostrich eggs has been described as a potential alternative concept to common animal testing using rats or mice [1–4]. This approach bears the advantage that, according to national and international legislation, research using eggs does not qualify as animal testing as long as all experiments are carried out before hatching [5–8]. Thus, elaborate application for permission to conduct an animal experiment as well as adequate animal housing, trained personnel and specific equipment is – at least in part – expendable.

Usually, chicken embryos are used for in-ovo imaging; however, this requires dedicated small animal imaging devices which represents a disadvantage regarding limited access [1, 2, 4]. A concept using substantially larger ostrich eggs and imaging devices commonly used in routine clinical examinations in humans has been published before [1, 2, 4]. Important questions have been answered regarding implementation in a nuclear medicine research facility, physiological embryo development on serial CT-scans and immobilization using narcotic gases in order to minimize embryo movement during scans, e.g., list-mode PET/CT [1, 2, 4, 9, 10]. Given these preliminary studies regarding ostrich-based imaging, the next step requires systematic description of well-known radiopharmaceuticals in this novel preclinical imaging model which exceeds the information known from previous studies [4].

Thus, this study aims at describing methods of intravenous injection in ostrich embryos and investigating normal biodistribution of F-18-FDG in ostrich embryos using a standard PET/CT system on development day (DD) 37. Data obtained from dynamic list-mode examinations over 60 min are quantified and interpreted regarding image quality and compared to data obtained from studies investigating rodents and chicken eggs. Additionally, effect of isoflurane narcotic gas on F-18-FDG distribution is assessed. The understanding of normal biodistribution of F-18-FDG is necessary in order to develop disease models (e.g., inflammation or tumor models).

## Materials and methods

### Ostrich eggs

Ostrich eggs were obtained from a local ostrich farm 15 km from the research facility between April and September. Artificial incubation was carried out using a multistage egg incubator (Sofie 3, Hemel, Verl, Germany) with constant incubation properties at 36.5°C and 25% air humidity as described elsewhere [2, 4]. If artificially incubated, ostrich eggs usually hatch after 42 days [11]. As it was a requirement to end all experiments before hatching, studies were performed on DD 37. This embryo study did not qualify as an animal research study according to the Federal German Animal Protection Act. Registration took place with the Office for Consumer Protection of the Thuringia State, registration number 22-2684-04-02-114/16. All experiments were carried out in compliance with German and international animal welfare legislation.

### Immobilization

In order to prevent motion artifacts during 60-min list mode dynamic PET/CT scan, a part of the ostrich embryos were exposed to narcotic gas isoflurane (Piramal Healthcare, Mumbai, India) using a standard vaporizer (Vapor 2000 Isofluran, Draeger, Luebeck, Germany) and a fix concentration of 6% which has been described effective for immobilizing ostrich embryos [1, 9]. For 60 min, isoflurane exposure was performed in a gas-tight container prior to PET/CT scan. Ostrich eggs were subsequently transferred to a working bench, preparing for vessel access.

### Vessel access

**FIGURE 1:**
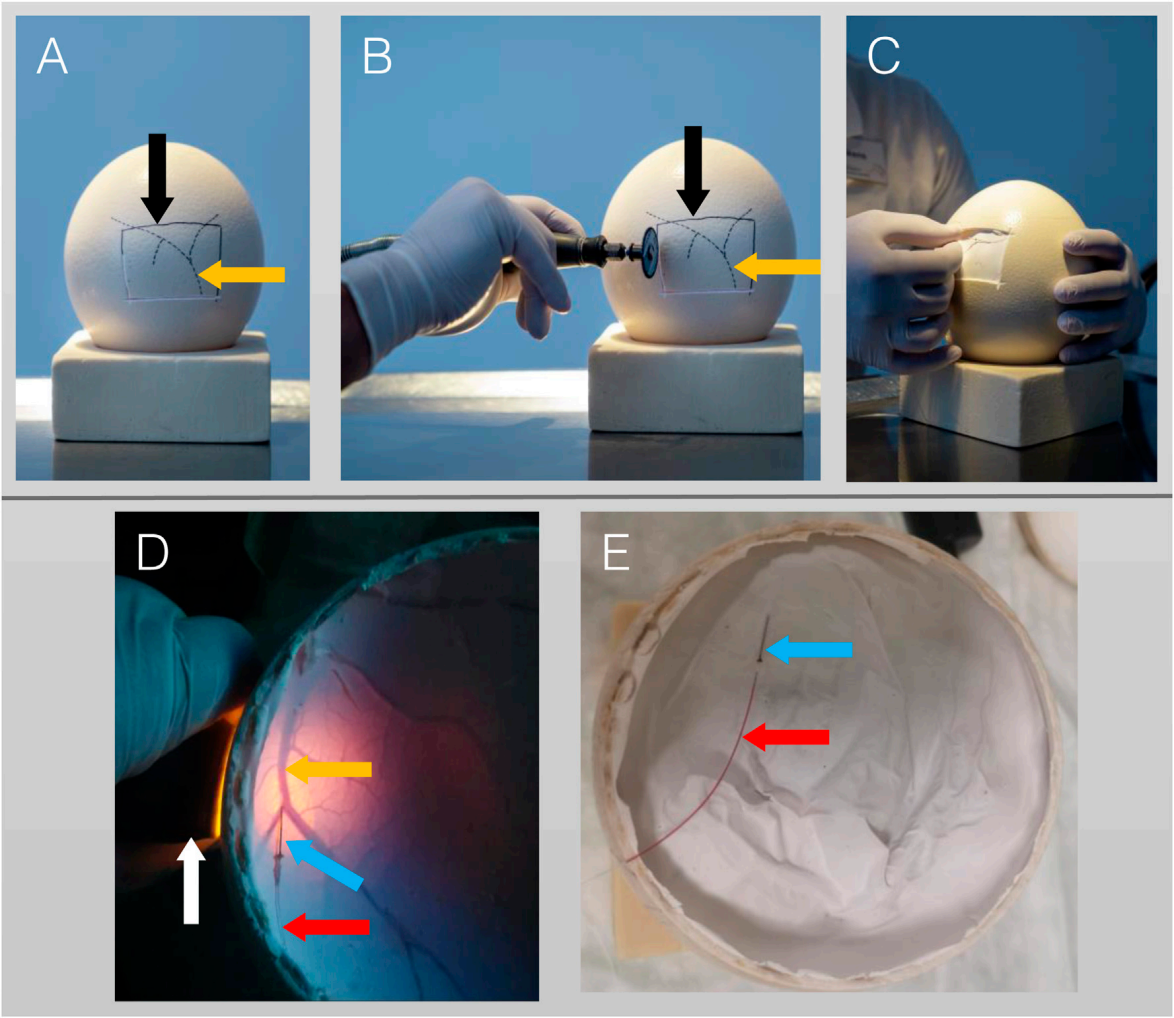
Procedure of vessel access on DD 37. **(A–C)**: Lateral access. **(A)**: Lateral view with vessels (orange arrow, dotted lines) which were identified on DD 25 by candling and marked with a pencil. The solid line (black arrow) represents a rectangular area of the eggshell to be removed. **(B)**: Cutting of the eggshell using a rotating cutter along the solid line. **(C)**: The rectangular area of the eggshell has been removed and cannulation of a small vessel is performed. **(D,E)**: Top access/Decapitation. **(D)**: After the whole eggshell at top of the egg (containing the air cell) has been removed, a candling light (white arrow) is placed laterally, illuminating the CAM-vessels (orange arrow) beneath the white egg shell membrane. Cannulation is performed using a 30 gauge cannula (blue arrow). **(E)**: Successful vessel access indicated by blood backflow into the catheter (red arrow).

Intravenous application of radiopharmaceuticals requires establishing a vessel access. Candling (diaphanoscopy-like illumination) of ostrich eggs was performed on DD 25 and DD 28, identifying faintly visible vessels of chorioallantois-membrane (CAM) located beneath the eggshell. Subsequently, the vessels’ location and course is marked on the eggshell using a pen. During later development stages, identification of CAM vessels is obscured by extended shadowing of the large ostrich embryo. On DD 37, part of the eggshell was removed using a rotating cutter (Dremel, Bosch Powertools B.V., Breda, Niederlande), either by windowing (removing a rectangular part of the eggshell; Figures 1A–C) or by decapitation (removing the whole eggshell at the end of the egg containing the air cell; Figures 1D,E). Subsequently, CAM vessel was punctured using a 30 gauge cannula (Sterican, B. Braun, Melsungen, Germany) (Figures 1D,E), connected to a polyethylene tube with an inner diameter of 0.28 mm (BD Intramedic, Fisher Scientific GmbH, Schwerte, Germany), and flushed with saline.

### Radiopharmaceutical

F-18-fluorodeoxyglucose (FDG) was obtained from Life Radiopharma f-con GmbH (Holzhausen an der Haide, Germany). 1-mL syringes were filled with approx. 2–10 MBq F-18-FDG and total volume of <0.5 mL. Pre-injection and post-injection syringe activity was measured using a standard dose calibrator (Isomed 2010, Nuvia Instruments GmbH, Dresden, Germany).

### PET/CT data acquisition and reconstruction; image analysis

After establishing a vessel access, PET/CT examination started using a standard scanner for clinical routine examinations in human patients (Biograph mCT 40, Siemens Healthineers, Erlangen, Germany). First, full-dose CT-scan (120 kV, 200 mAs, increment 0.3 mm, slice thickness 0.6 mm, filtered back projection) was acquired for attenuation correction purposes and anatomic co-registration. List-mode dynamic PET was started immediately after injection of F-18-FDG and data were acquired for 60 min. PET data reconstruction was performed using iterative technique (4 iterations, 12 subsets, matrix 400, Gaussian filter, zoom factor 2 and optimized proprietary reconstruction mode True X (Siemens Healthineers) comprising point spread function and ordered subset expectation maximization (OSEM) algorithms. List-mode data were reconstructed in 120 30-s-timeframes as well as additional 10-s frames during the first 2 minutes. Image analysis and quantification was performed using proprietary software (syngo.via, version VB50BHF02, Siemens Healthineers). In order to quantify activity distribution, spherical volume-of-interests (VOIs) were drawn and activity was expressed as standardized uptake value (SUV), kBq/mL as well as relative injected activity per mass (IA%/g). For each organ/compartment, two VOIs were drawn and the mean value of both measurements was used for further analysis. IA%/g was chosen for comparison of PET and *ex-vivo* biodistribution data because well counter measurements cannot be expressed as SUV. Regarding dynamic activity distribution correct VOI position was manually verified in each timeframe. Patlak plots were derived from dynamic PET data using a blood-derived input function.

### *Ex-vivo* biodistribution

Quantification of activity distribution of different ostrich organs was verified by *ex-vivo* measurements. After PET/CT examination, ostrich embryos were sacrificed by i.v. injection of 500 mg sodium pentobarbital. Organs (i.e., brain, heart, liver, ventriculus, intestine, kidneys) and fluids (i.e., yolk, blood) were collected and specific activity was measured using a standard well counter (Isomed 2100, Nuvia Instruments).

### Statistics

Data analysis and descriptive statistics were performed using Excel (Microsoft Excel 2016, Microsoft Corporation, Redmont, WA, United States). Values were expressed as mean and standard deviation was given, if applicable. Correlation was calculated using Spearman’s Rho Correlation Test and p-values < 0.05 were considered significant. Bland-Altmann-plots were analyzed in order to exclude bias.

## Results

**FIGURE 2:**
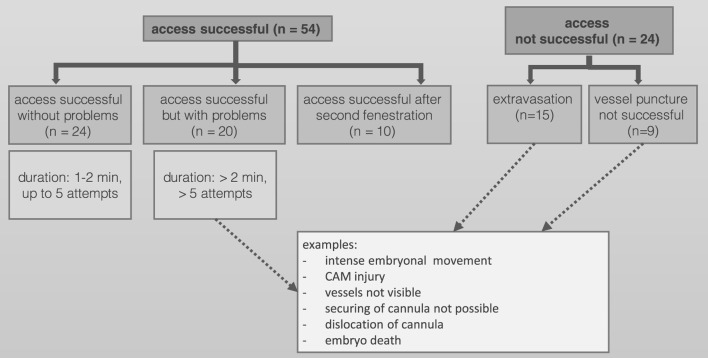
Success of vessel access and reasons for partial or complete failure.

A total of 339 ostrich eggs were obtained from a local ostrich farm. 78/339 (23.0%) showed fully developed ostrich embryos on DD 37 and were prepared for PET/CT imaging. Success of vessel access and reasons for partial or complete failure are shown in Figure 2. In total, 54/339 ostrich embryos were available for dynamic PET/CT imaging (60-min list mode) with different radiopharmaceuticals. Regarding normal distribution of F-18-FDG, twelve ostrich embryos were investigated after exposure to isoflurane. Four different ostrich embryos served as control group and were not exposed to isoflurane before PET/CT examination.

### Visual image analysis

**FIGURE 3:**
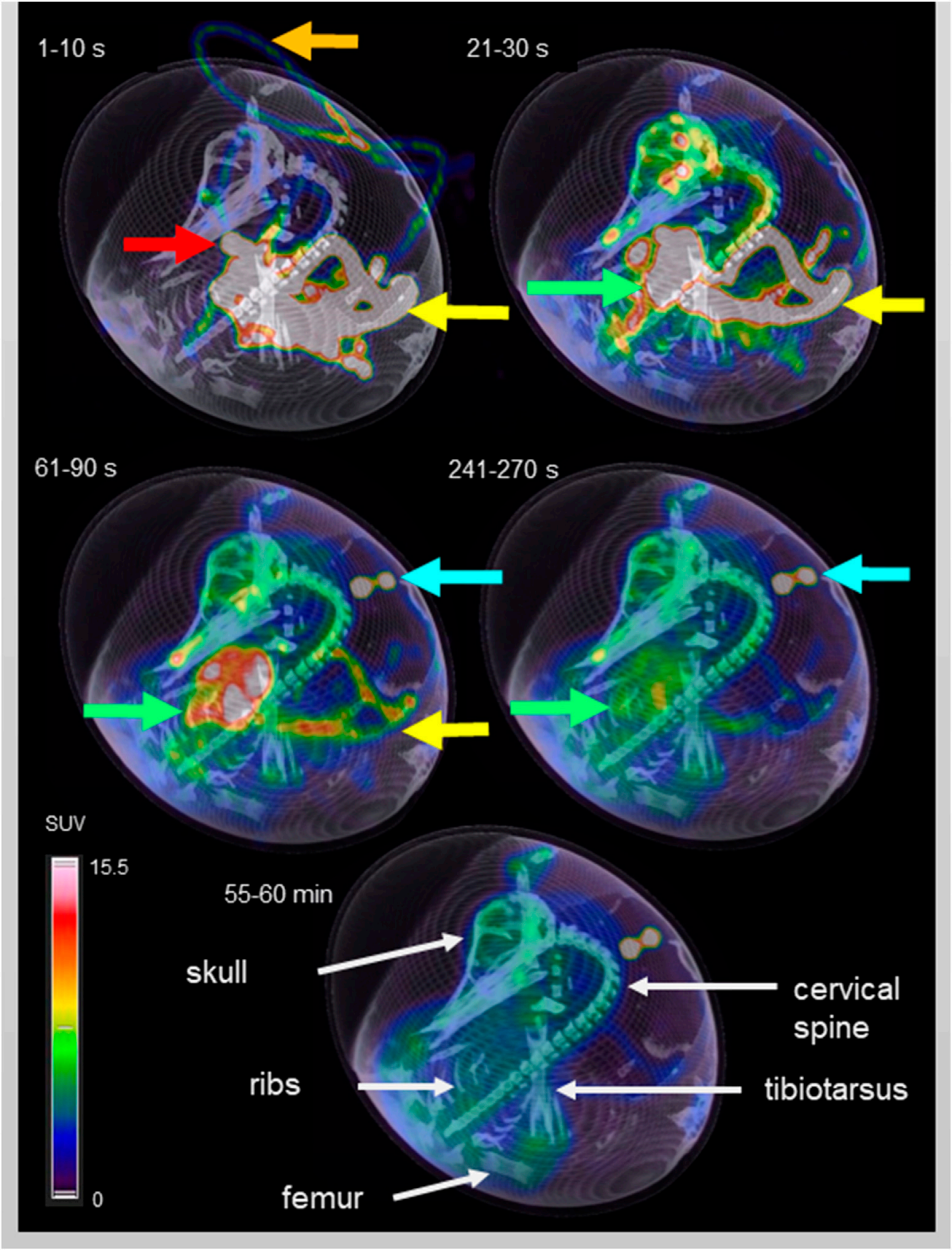
Dynamic PET/CT scan of an ostrich egg on DD 37. Fusion imaging of maximum intensity projection (MIP, PET) and virtual rendering technique (VRT, CT) was chosen for three-dimensional visualization. Timeframes in the upper left corner of each image represents the time after injection (p.i.) of 10 MBq F-18-FDG. In the first timeframe (1–10 s p.i.) the activity in the plastic tube is visible (orange arrow), caused by the injection. Additionally, the vitelline vein is depicted (yellow arrow) in which the blood flows from the CAM to the embryo. Due to high specific activity within a small volume, the embryo’s vessels show an intense signal. Considering later time points, it is possible to identify an area of high uptake in the embryo’s thorax during the first timeframe which decreases over time and represents the heart/blood activity (red arrow). The second image (21 – 30 s p.i.) shows accumulation of activity within the whole embryo, mainly in the vessels and starting in soft tissue. Activity in the vitelline vein (yellow arrow) is decreasing and the liver (green arrow) represents the organ with highest activity accumulation. The following two images (61 – 90 s p.i. and 241–270 s p.i.) are characterized by steady decrease of blood and liver activity, and increasing accumulation in soft tissue instead. In general, a more homogeneous activity distribution is observed compared to the early timeframes. The blue arrow marks activity which is located outside of the egg, caused by residual syringe activity placed next to the egg after application of F-18-FDG. The last image gives an overview of the activity distribution 55 – 60 min p.i. using the same thresholds for MIP-imaging. FDG-uptake is visible in the brain and in epiphyseal plates (see also Figure 4). White arrows mark anatomical structures for anatomic co-registration of activity distribution.

**FIGURE 4:**
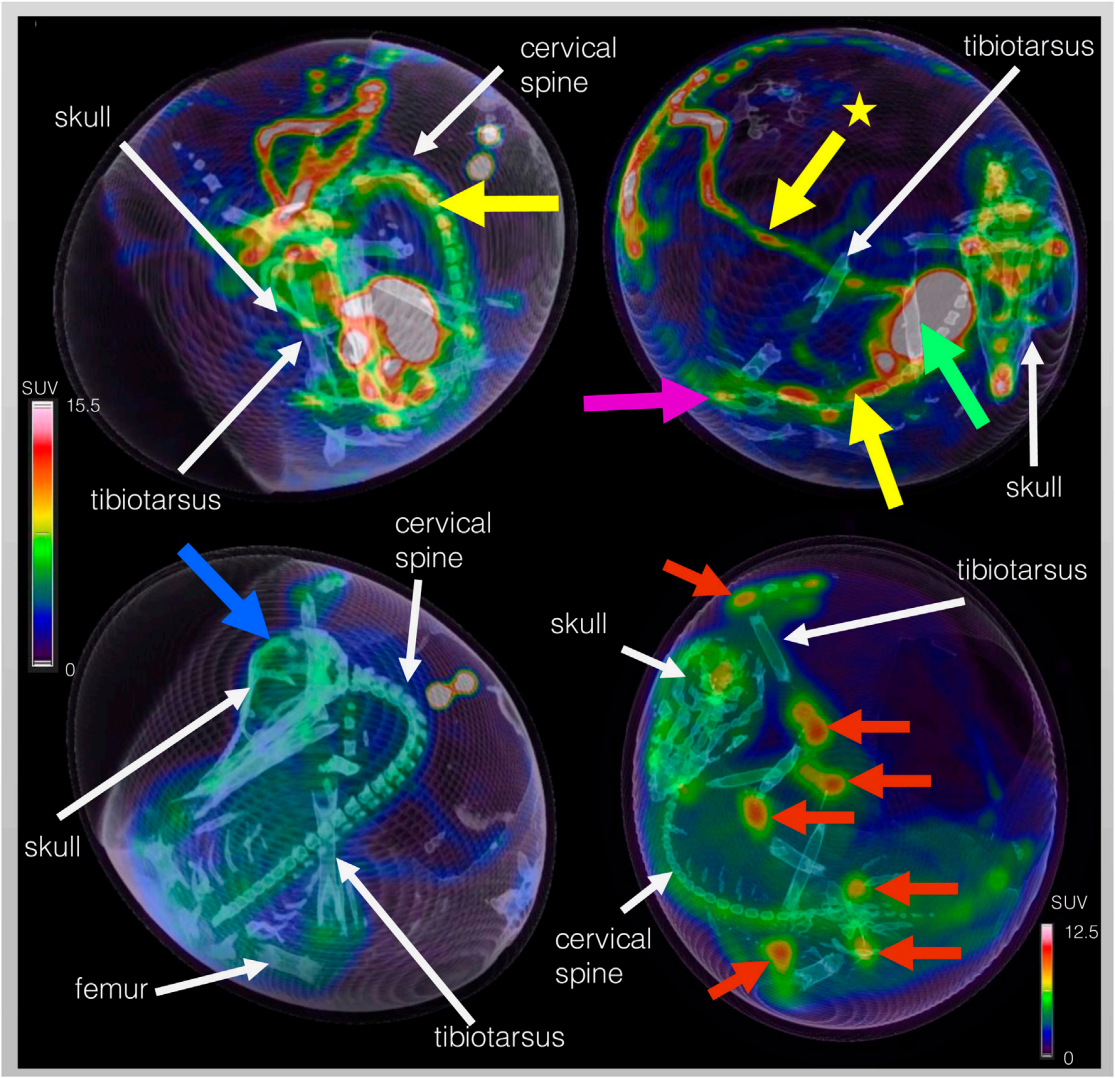
Dynamic F-18-FDG PET/CT scan of the same ostrich embryo as depicted in Figure 3 but with focus on representative images of organs and structures. Embryo vessels are shown in the two images of the top row at 31 – 40 s p.i. The yellow arrow marks the long carotid arteries (upper left image) and the abdominal aorta (upper right image) inferior to the liver (green arrow). The yellow arrow with star points at the extraembryonal vitelline vein. In the upper right image, activity within the kidneys is visible (pink arrow). Brain activity is marked with a blue arrow in the lower left image (40 min p.i.). In order to depict activity distribution in the epiphyseal plates (red arrows) of the lower extremity (femur, tibiotarsus, tarsometatarsus), scaling was adjusted. White arrows mark anatomical structures for anatomic co-registration of activity distribution.

Five different time points of dynamic list mode PET/CT are shown in Figure 3, representing activity distribution over time. Additionally, images of different organs/structures are shown in Figure 4.

### Quantification

**FIGURE 5:**
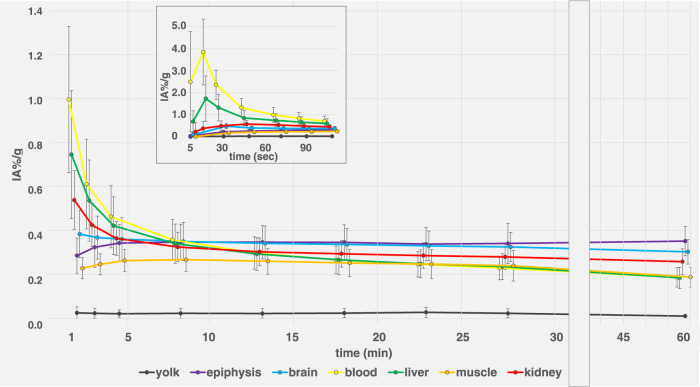
F-18-FDG biodistribution of 12 ostrich eggs in different organs and compartments (yolk, epiphyseal plates, brain, blood, liver, muscle, kidney) over 60 min, assessed via VOI measurements on PET/CT. X-axis was adjusted for late time points (>30 min) for better visualization.Insert graph on top shows first 120 s for assessment of early distribution effects, specifically blood curve (yellow). Data are expressed as relative organ activity per injected activity (IA%/g). Whiskers represent standard deviation. For clarity purposes, time points were spread in order to allow for delineation of data point whiskers. At 60 min p.i., epiphyseal plates (purple line) and brain (blue line) show highest uptake. Yolk shows consistently low FDG uptake over time.

**FIGURE 6:**
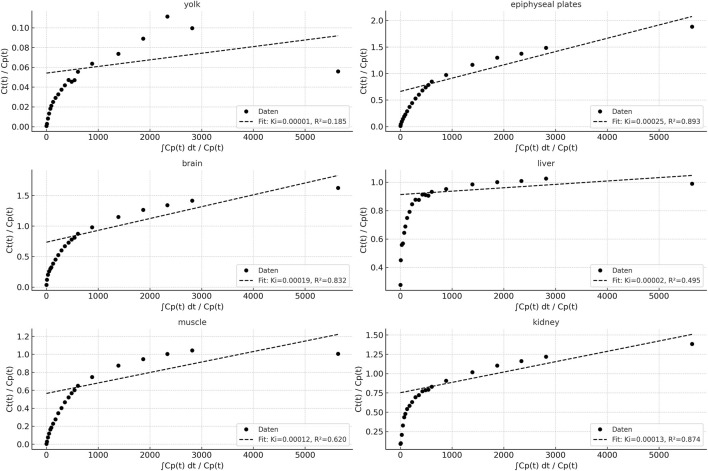
Patlak plots for six different organs (yolk, epiphyseal plates, brain, liver, muscle, kidney), derived from dynamic PET data using a blood-derived input function. The x-axis represents the normalized integral of the input function, while the y-axis displays the normalized tissue concentration. For each organ, linear regression was applied from t = 135 s onward, excluding the final time point (3600 s) to improve model robustness by reducing the influence of potential noise and late-phase redistribution.

**FIGURE 7:**
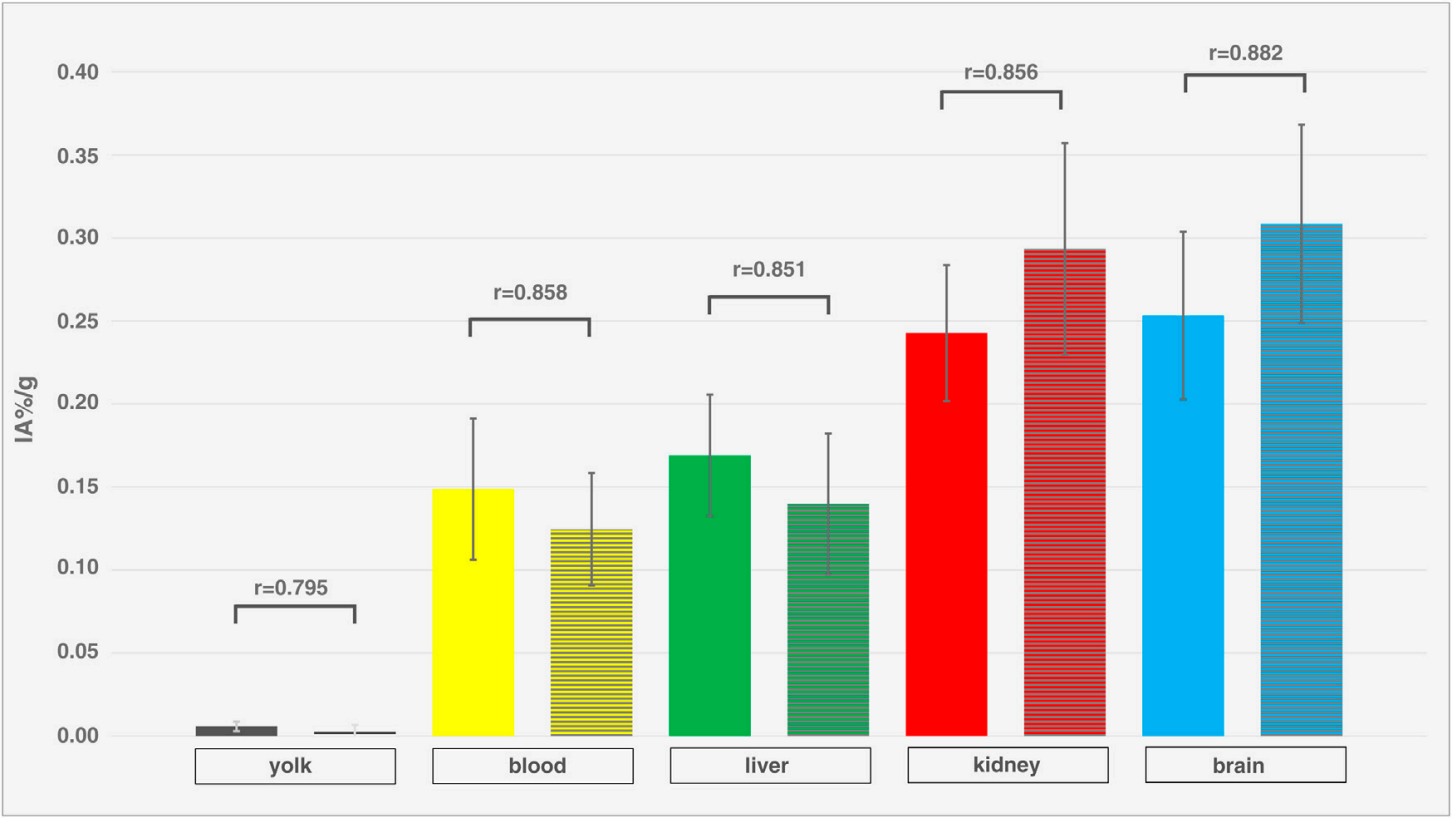
Comparison of *in-vivo* and *ex-vivo* measurements. Organ activity of different compartments and organs (yolk, blood, liver, kidney, brain) of different ostrich embryos (isoflurane, n = 12), assessed via VOI measurements on PET/CT (left columns, without texture) 55 min p.i. and *ex-vivo* measurements (right columns, with texture). Whiskers represent standard deviation. Data are expressed as relative organ activity per injected activity (IA%/g). R-values were calculated using Spearman’s Rho and p-values are < 0.0001 for all compartments and organs. As all R-values are > 0.8, a very strong correlation is present between PET and *ex-vivo* measurements indicating sufficient reliability of PET quantification.

Dynamic activity distribution over time is shown in Figures 5, 6 Organ activity assessed 55 min p.i. via F-18-FDG-PET/CT and *ex-vivo* activity showed comparable results (Figure 7). Analyses using Bland-Altman plots revealed all data within 1.96-times standard deviation without significant overestimation or underestimation (data not shown). Highest uptake was found in epiphyseal plates (0.36 ± 0.06 IA%/g; range 0.29–0.48 IA%/g).

### Effect of isoflurane

**FIGURE 8:**
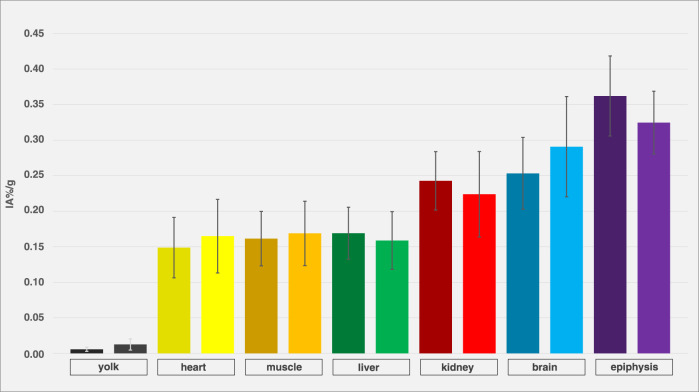
Comparison of embryos with and without isoflurane exposure. Organ activity of different compartments and organs of different ostrich embryos after exposure to isoflurane (left columns, dark-colored, n = 12) and without exposure to narcotic gases (right columns, light-colored, n = 4), assessed via VOI measurements on PET/CT 55 min p.i. Whiskers represent standard deviation. Data are expressed as relative organ activity per injected activity (IA%/g). Using Shapiro-Wilk test and Kruskal-Wallice test, no significant differences were observed.

Figure 8 shows organ activity in ostrich embryos after exposure to isoflurane (n = 12) and without exposure to narcotic gases (n = 4). No significant differences were observed.

## Discussion

This study describes normal biodistribution patterns of F-18-FDG in ostrich embryos 5 days before hatching.

### Strengths of in-ovo imaging using ostrich embryos

The idea to use ostrich embryos arose from two specific limitations at the research facility: First, the lack of a small-animal imaging device for biodistribution studies, and second, prolonged time until receiving permission for classic animal testing using rodents. These limitations were addressed by using ostrich embryos which are large enough to be investigated in regular imaging devices used in humans and which do not qualify as animal testing under a legal view. The use of avian eggs, specifically chicken eggs, is widely distributed; however, mostly focusing on well-vascularized CAM as a biomembrane, allowing for tumor cell growth and thus, monitoring anti-neoplastic substances. The embryo itself usually is not in the focus of CAM-experiments. This is an unrealized potential as the embryo offers interesting advantages over rodents: First, avian embryos only need warmth and oxygen for regular development. All other resources (nutrition, water) are inside the eggshell, thus, no feeding is required. Second, during the second half of breeding, all organs are fully developed and thus, the whole body may be investigated. In contrast, CAM only focuses on the properties of the implanted tumor or the CAM vessel reaction to the tumor. The limitations of using ostrich embryos are addressed below.

### Artificial incubation and vessel access

Several steps are necessary in order to obtain a viable ostrich embryo on DD 37 for PET/CT imaging. First, eggs have to be fertilized which varies according to season, weather conditions and ostrich hens’ and rooster’s health [12–14]. Fertilized eggs are identified as early as DD 13 by candling and are continuously stored in the incubator while unfertilized eggs are removed and discharged [12, 14]. Second, during the following development steps, death-in-shell (DIS) can occur which also reduces the number of viable ostrich embryos [13]. Third, successful vessel access is required for i.v. injection of radiopharmaceuticals. After these steps, in the current study a total of 16% of initially obtained ostrich eggs contained viable embryos available for PET/CT imaging. Success rates for artificial breeding in ostrich farms vary from 40 to 70% which is higher than in this study [12, 15, 16]. This is attributable to distinct low rate of fertilization in our research facility in 2021 as previous years revealed fertilization in 29, 38 and 62% of ostrich eggs, respectively [14]. As it is difficult to improve fertilization rates *per se*, effectiveness of in-ovo imaging using ostrich eggs could be increased by improving methods of artificial breeding, thus avoiding DIS, and establishing vessel access, latter bearing higher potential with increasing handling experience [17, 18]. Also, ostrich egg supply needs to be considered. For this study, the ostrich eggs were provided by one farm. A diversification of egg supply could add to improved artificial breeding success in years of low fertilization rates at one farm.

### Visual image analysis

*In-vivo* biodistribution of F-18-FDG in ostrich embryos was found as expected. Image example of one ostrich embryo is shown in Figures 3, 4 with early tracer distribution in cardiovascular system, followed by liver accumulation and subsequent soft-tissue enhancement. At 55 min, epiphyseal plates and brain are the areas of highest tracer accumulation. Notably, soft-tissue clearance over time in ostrich embryos is less than biodistribution pattern described in rodents and humans. This is likely caused by lack of sufficient renal tracer excretion in ostrich embryos which rely on allantoic metabolite deposition instead of urinary excretion in adult animals and humans [19, 20]. Recently, comparable results have been described by Smith et al. for chicken embryos on DD 14 [18]. Earlier publications report on biodistribution of different radiopharmaceuticals, including FDG, in chicken embryos using a small-animal PET/CT system, also showing rather high soft-tissue accumulation of FDG [21]. Missing renal excretion of radiopharmaceuticals is known from patients with chronic kidney failure depending on dialysis. In most cases, the results of F-18-FDG-PET/CT are not significantly hampered and the clinical question is answered as accurately as in patients with normal renal function. Thus, high soft-tissue tracer accumulation is not regarded as a significant limitation of in-ovo imaging using ostrich eggs.

Wu et al. described brain FDG uptake in mice over 60 min [22]. The results obtained in the current study suggest that brain uptake in ostrich embryos is less evident than reported for humans and awake mice. This might be attributable to three main factors: First, brain function in ostrich embryos is not yet fully developed before hatching, thus low glucose metabolism can be assumed. Gradually increasing cerebral glucose metabolism has been described for developing rats during pre- and postpartal period [23] which is supported by analyses that the developing mammalian brain uses different substrates (e.g., ketones, fatty acids) and glucose, whereas the adult brain solely relies on glucose for energy supply [24]. Second, apart from cerebral base rate glucose metabolism, stimuli and sensations are low in a concealed egg shell, thus contributing to low brain activation. The brain of chicken embryos showed variable FDG-uptake in a study performed by Balaban et al. investigating different brain regions and describing active and inactive brain states [25]. Third, avian neurons have been described to consume three times less glucose than mammalian neurons; however, focusing on adult organisms and thus being comparable only with limitations to embryos development stages [26].

Out data show that glucose metabolism in the liver is lower than in brain, kidneys and epiphyseal plates. In humans, GLUT1 is the main glucose transporter in fetal liver, ensuring insulin-independent glucose uptake via placental circulation [27], suggesting high glucose uptake. After birth and following enteral feeding, GLUT1 is downregulated and GLUT2 expression gradually increases. Furthermore, gluconeogenesis and glykogenolysis mature, stabilizing blood glucose levels [27]. Metabolic analyses of post-hatch chicken show early presence of glycolysis and gluconeogenesis as well as reliance on amino acids and fatty acids which are stored in the yolk, resembling the processes that occur in mammals. After hatching, during a time period of 20 days, metabolism changes in order to effectively utilize fed nutrients () [28] and – as in mammals - GLUT2 is also expressed [29]. FDG-PET/CT show similar distribution patterns in chicken embryos compared to the presented data [21]. Regarding ostriches, little is known about the embryonic hepatic glucose metabolism but data derived from chicks and adult ostriches show comparative blood glucose levels, increasing with the individual’s age and development [30].

Tracer accumulation of FDG in epiphyseal plates is also known from examinations in children and indicates high glucose metabolism in sites of rapid bone growth with higher SUV-values for younger age groups [31, 32]. This has also been described for in-ovo imaging using chicken embryos [21, 33].

Regarding PET/CT imaging in humans, recent technological developments feature large scanners with multiple ring detectors creating a long axial field of view (e.g., uEXPLORER, United Imaging Healthcare; Biograph Vision Quadra, Siemens Healthineers). Liu et al. investigated normal biodistribution of FDG in healthy volunteers using scanners of this type [34]. One evident difference between humans and ostrich embryos is predominant tracer distribution within lung tissue during the first 5 minutes, representing physiological blood flow in pulmonary vascular system. Due to embryonic circulation bypassing pulmonary vessels, lung tissue was not identified on FDG-PET studies in ostrich embryos.

Notably, no individual showed myocardium uptake. Human myocardium is known for variable inter- and intraindividual FDG uptake, depending on the main source of energy supply at the moment of FDG administration. As both fatty acids and glucose are suitable as energy substrates for myocardium, both metabolic states can be found in patients [35]. Insulin increases cardiac FDG-uptake in mice [36] and humans [37]. Bencurova et al. reported on biodistribution of FDG in chick embryos on DD 16-18 describing little FDG uptake within the heart, however, without exactly stating whether myocardium uptake or blood-pool-activity was regarded as the source of tracer accumulation [33]. However, Souza et al. described variable cardiac FDG uptake related to myocardium in a small study investigating adult parrots, indicating variability in avian species [38]. Up to date, there are no comparable cardiac imaging studies using FDG-PET/CT in chicken or ostriches but Kutchai et al. described *ex-vivo* experiments assessing high glucose uptake in chick embryos during early development with decreasing uptake over time [39]. –This is supported by autoradiography experiments by Kostreva who investigated C-14-deoxyglucose chicken heart and stating high glucose dependence of embryonic myocardium of different species [40] More invasive experiments as conducted currently could contribute to the understanding of ostrich embryo myocardium uptake, i.e., co-injection of insulin or glucose.

### Quantification

Visual description of FDG biodistribution is supported by quantification using VOI-measurements in PET over time (Figures 5, 6) as well as *ex-vivo* measurements after organ collection and activity measurement using a well-counter (Figure 7).

Tissue activity curves using the Patlak model provide an approximation for tracer kinetics of various tissues and compartments. High linearity was observed in brain and epiphyseal plates (R^2^ > 0.98), indicating strong irreversible tracer uptake and model conformity. The influx rate constant Ki reflects organ-specific tracer accumulation rates, with the kidney and liver showing higher Ki values, consistent with their metabolic/excretory roles. The yolk and muscle curves exhibited flatter slopes and lower R^2^ values, suggesting limited tracer trapping or predominantly reversible kinetics in these tissues.

Organ time activity curves in humans show comparable values to data obtained in ostrich embryos in this study [41–43]. Two different ways are generally used for describing radiopharmaceutical uptake in PET studies: IA%/g and SUV. While IA%/g is independent from body weight and total volume, SUV uses body weight as a factor which allows for comparison of species with different weight. Both methods bear advantages and disadvantages that have to be considered when comparing FDG uptake within different species. In this study, IA%/g was used for data description as it is suitable for both PET and *ex-vivo* well counter measurements, allowing direct comparison of both. Most preclinical studies focus on IA%/g and ID%/g (relative injected dose per mass), respectively [44, 45], and use these parameters for imaging and *ex-vivo* well counter measurements as well. SUV is a parameter commonly used in clinical routine to assess radiopharmaceutical uptake in patients. It offers a sufficiently robust, yet straightforward and reproducible method for quantifying tracer accumulation, for example, in tumor lesions [46, 47].

Rodent and in-ovo PET-imaging have been described to show similar uptake of reference regions using PSMA-ligands [48]. In order to contextualize the FDG-uptake values derived from ostrich embryos and described in this study, Table 1 gives an overview of data published on different species and FDG normal distribution (Table 1). This data also supports visual quantification in terms of soft tissue clearance: In ostrich embryos, levels of F-18-FDG accumulation are similar for liver and muscle tissue, whereas lower glucose metabolism is described for rodent muscle tissue than for rodent liver tissue. Table 1 also indicates the need for careful interpretation of activity quantification when species are compared because both SUV and IA%/g show substantial differences (Table 1).

**TABLE 1:** Overview of FDG uptake in different organs and different species.

Animal model	body weight	Brain				Liver				Muscle			
		organ weight (g)	SUV	IA%/g	reference	organ weight (g)	SUV	IA%/g	reference	organ weight (g)	SUV	IA%/g	reference
Chicken embryo (in-ovo)	60 g (egg) 20 g (embryo) on DD 18	0.89 g	-	4–8	[18, 21, 33, 49]	0.6 g	-	4–8	[21, 50]	-	-	2.5	[21]
Mouse	20–25 g	0.5 g	1.2	4–10	[41, 51–53]	1.5–3 g	0.5	1.0–4.1	[54–56]	-	1.5	0.6–2.2	[54–56]
Rat	170 – 290 g	1.7–2.0 g	1.4–1.8	0.4	[43, 57, 58]	12–19 g	0.6–1.0	0.31 +/− 0.16	[59, 60]	-	0.3	0.08 +/− 0.02	[61]
Ostrich embryo (in-ovo)	1,200 g (egg) 500 g (embryo) on DD 37	3.7 +/− 0.4	4.3 +/− 1.3	0.29+/− 0.07	This study	6.1 +/− 1.1 g	2.8 +/− 0.5	0.20 +/− 0.04	This study	-	2.4 +/− 0.4	0.2 +/− 0.04	This study
Rabbit	2.5–5 kg	9 g	1.8	0.01	[62, 63]	80 g	2.5	-	[62, 63]		0.2	-	[62, 63]
Miniature Pig	10–20 kg	100 g	1.1–3.1	0.03	[64, 65]	320 g	1.15	-	[64, 65]		0.3	-	[65]
Human	75 kg	1400 g	5–15	0.001–0.005	[41, 66]	1,500 g	2.0–2.4	-	[67, 68]		0.4–1.4	-	[69]

Abbreviations: DD, development day; SUV, standardized uptake value; IA–injected activity.

As quantification relies on successful intravenous application of radiopharmaceuticals, special attention has to be paid to paravasation which particularly influences PET quantification in dynamic studies [17, 18, 70].

### Effect of isoflurane

This study also assessed the effect of isoflurane on various organs and compartments. No significant differences were found for yolk, blood, muscle, liver, kidney, brain and epiphyseal plates. This result was unexpected as isoflurane usually reduces brain glucose metabolism and thus, FDG uptake, which has been reported for mice and other mammals [71] and is also known for PET examinations in humans after sedation [72]. Also, changes in FDG-metabolism of other organs, e.g., liver, kidneys and muscles have been reported after application of isoflurane [54].Thus, the effect of isoflurane on glucose metabolism of ostrich embryos as well as other anesthetic or muscle relaxing agents needs to be further investigated. The immobilization effect of isoflurane anesthesia on ostrich embryos was not in the scope of this study and has been described elsewhere [9, 14].

### Limitations

This study has limitations that need to be considered when interpreting the results. First, the number of ostrich embryos is rather small (n = 16) and thus, quantification results are not as reliable, limiting the statistical power of this study. The confidence intervals of biodistribution data (Figures 5, 7) and comparison with a control group (Figure 8) show high variation which could be improved by including more individuals per group. Efforts have to be made in order to increase artificial breeding success by early dismissal of non-fertilized and bacterially infected eggs as well as increasing the number of eggs orderd from the farm. Second, dynamic data was evaluated only using VOI-measurement technique in PET. Invasive arterial input functions were not considered as it has not been established to draw arterial blood in-ovo. Third, regarding reliability of PET-based quantification, signal-to-noise ratio (SNR) was not quantitatively assessed. The spatial resolution of the clinical PET/CT scanner (Biograph mCT 40; FWHM 4.4 mm) used in this study is inherently limited compared to small-animal systems (e.g., Siemens Inveon PET; FWHM 1.2 mm), potentially introducing partial volume effects, especially in small embryonal structures. This may have impacted quantification accuracy in organs difficult to delineate (e.g., kidney). In order to verify correct PET quantification, VOI data were compared to *ex-vivo* measurements, producing concordant results (Figure 7). This leads to the conclusion that PET quantification is sufficiently correct.

Fourth, direct comparison of ostrich embryo and similar-sized established animal models using the same scanner was not performed. This setup would allow for even more detailed comparison of both imaging models. Fourth, although there are numerous ostrich farms in Germany [12], ostriches lay eggs only from April to September, which limits the availability to these seasons. This represents a disadvantage compared to chicken eggs which offer year-round access and has to be considered in experiment planning. The use of eggs of other ratites (e.g., emu, dromaius novohollandiae) might possibly overcome this availability gap as they are available from December to March [12, 58].

### Future direction and outlook

Despite the aforementioned limitations of this study and the concept of in-ovo imaging using ostrich eggs, this approach has the potential to be integrated into the landscape of preclinical research, specifically imaging studies. Of course, the results presented in this article need further investigation and verification (e.g., blocking studies evaluating tracer uptake specificity, sophisticated quantification using invasive arterial input functions) as well as expanding the field of application to other tracers. In order to be used in research projects, the normal biodistribution described in this study is not sufficient; thus, the development of disease models (e.g., tumor models, inflammation models) is crucial. This allows for interventional studies, testing new radiopharmaceuticals (aimed at tumor or inflammation) or anti-tumor (anti-inflammation) treatment agents and visualizing the therapeutic effect by preclinical imaging, i.e., F-18-FDG-PET/CT. In rodents, subcutaneous injection of substances is well established and could be transferred to ostrich embryos as well. These substances comprise tumor cells (either tumor cell lines or patient-derived tumor cells or organoids), inflammation agents (Carrageen, bacteria), and more. Establishing tumor and inflammations models in ostrich embryos requires several steps: First, it is necessary to determine the best way to inject a substance (i.e., tumor cells, inflammation agents) into the embryo without harming vital structures, requiring visual guidance (either via ultrasound or CT) and identification of optimal injection site (i.e., subcutaneous, intraperitoneal, yolk sac). Second, selection of optimal parameters (concentration, incubation time) is crucial to grow tumors and induce inflammation, respectively. Third, experiments are necessary to prove adequate therapy response, e.g., the size of a tumor in response to an established chemotherapy protocol with a control group without antineoplastic treatment. Regarding inflammation, response of a local infection to an established antibiotic regimen should be part of experiment planning. Fourth, appropriate PET radiopharmaceuticals need to be selected for tumor imaging (FDG or Ga-68-FAPI (fibroblast activating protein inhibitor) targeting cancer associated fibroblasts) and inflammation imaging (FDG or Ga-68-Pentixafor targeting chemokine signals (CXCL12/CXCR4 pathway), respectively. This allows for assessing the therapeutic effect via PET imaging.

In addition to utilizing the embryo as an experimental model, CAM assays using chicken eggs is an established alternative for animal testing, growing tumors and inducing inflammation on this highly-vascularized biomembrane [18–20]. Transfering this knowledge from chicken CAM to ostrich CAM has already been reported [10]. Summarizing, once disease models have been developed, preclinical imaging using ostrich embryos might represent a contribution to 3-R- principles reducing the required number of fully developed animals.

### Conclusions

This study describes PET imaging using ostrich embryos, representing an alternative imaging model for preclinical imaging. Normal biodistribution of F-18-FDG in ostrich embryos using a standard PET/CT system for humans was mainly found as expected with highest uptake in brain and epiphyseal plates which is comparable to results on rodents and chicken embryos. Isoflurane anesthesia was applied to part of the individuals in order to reduce motion artifacts and revealed no significant differences regarding organ uptake. The results of this normal distribution study allow for interpretation of future disease models (e.g., inflammation, tumor) in ostrich embryos using FDG as radiopharmaceutical.

## Data Availability

The raw data supporting the conclusions of this article will be made available by the authors, without undue reservation.
